# Unveiling antimicrobial and anticancerous behavior of AuNPs and AgNPs moderated by rhizome extracts of *Curcuma longa* from diverse altitudes of Himalaya

**DOI:** 10.1038/s41598-020-67673-4

**Published:** 2020-07-02

**Authors:** Mamta Sharma, Monika, Pankaj Thakur, Reena V. Saini, Rajesh Kumar, Enza Torino

**Affiliations:** 1 School of Biological and Environmental Sciences, Shoolini University, Solan, 173212 India; 2 School of Chemistry, Shoolini University, Solan, 173212 India; 3 Himalayan Center of Excellence in Nanotechnology, Shoolini University, Solan, 173212 India; 4 School of Biotechnology and Applied Sciences, Shoolini University, Solan, India; 5 School of Physics and Materials Sciences, Shoolini University, Solan, India; 6 Center for Advanced Biomaterials for Healthcare, Italian Institute of Technology, 80125 Naples, Italy

**Keywords:** Plant sciences, Nanoscience and technology

## Abstract

Conservative remedies have a gray history worldwide and these provide productive and pertinent tools to tackle ailments. Also, the high altitude areas of Indian Himalayas with their wealthy biodiversity anchorage around 2000 plant species. Ensuing study demonstrates the synthesis of Silver (Ag) and gold (Au) nanoparticles (NPs) and utilizes one of the medicaments *Curcuma longa* of Indian Himalayas collected from different altitudes. For the same, turmeric rhizome extracts have been prepared from the aforesaid medicament and its anticancer activity and antimicrobial potential have been evaluated. Formation of Ag and Au nanoparticles was realized via UV–Vis spectroscopy and transmission electron microscope (TEM) confirmed size of the NPs. Antibacterial activity has been checked against *Bacillus subtilis* and *Escherichia coli.* The anticancer prospective has been observed against A549 and PC3 cell lines of both Au and Ag NPs and the cytotoxicity on PC3 and A549 cell lines was assessed using MTT assay. Results revealed higher amount of biochemicals, antibacterial and anticancer activity in Ag and Au NPs synthesized from rhizome extract collected from highest altitude. For the first time impact of altitudinal variations on phytochemicals and nanoparticles has been reported which have significant effect on its antimicrobial and anticancerous activity.

## Introduction

The altitudinal variations are one of the interesting aspects and may prove supportive in understanding the medicinal impacts of various plant species. Biosynthesis of the secondary metabolites in medicinal plants is not only genetically controlled, but is affected by biotic and abiotic stresses as well^1^. In different ecological niches, plants behave differently in terms of biochemical aspects in order to better adapt to their environment. This broad range of environmental factors across altitude thus affects both, the chemical-composition and ultimately the survival of medicinal plants in such regions. Ecological conditions affect secondary metabolites or compounds that plants produce, which are habitually basis for their medicinal activity^2^. Plant adaptation at higher altitude is regulated by avoiding and overcoming stress conditions by means of various physiological and biochemical mechanism. The aforesaid mechanisms include production of secondary metabolites, oxidative stress and evolution of resistance-conferring genotype that produce ecologically adapted phenotypes. Altitudinal variation consequences on phytochemicals, secondary metabolites profiles and bioactivities of higher plants has not been well explored. Even though plant products in different forms such as pastes and powder, etc. from various species of Indian Himalayas are still frequently being used by rural habitants for treating various infirmities. However, scientific validation for probable use of these medicaments for treatments of various diseases is exceedingly significant and need more exploration.

The chemical synthesis of nanoparticles is a preferred method, which encompasses the use of toxic chemicals as reducing, capping and stabilizing agents that may lead to the formation of harmful and unsafe non environmental friendly byproducts. The need for eco-friendly and non-toxic techniques for the synthesis of nanoparticles lead to an increasing interest in the biological protocols which require no use of harmful chemicals and lack in the formation of toxic byproducts^1,2^. Green synthesis of nanoparticles assign improvement over chemical and physical methods as it is easy, cost effective, eco-friendly and there is no need to use high energy, pressure, temperature and harmful chemicals^3^. The plant mediated synthesis of nanoparticles found to be suitable among different green synthesis methods as it favors to the formation of stable nanoparticles in less time^4^. Medicinal plants contain various phytochemicals which possess high therapeutic values provide better platform for the synthesis of nanoparticles as they are nontoxic and also provide natural capping agent^5^. Among the inorganic nanoparticles, gold and silver nanoparticles provide superior material properties with functional versatility. Silver nanoparticles, in particular, have attracted increasing interest because of the unique properties (e.g., size and shape depending optical, electrical and magnetic properties), which can be explored in antimicrobial applications, biosensor materials, catalytic applications, cosmetic products, electronic components and non-linear optics^6,7^_._ Gold and silver nanoparticles (Au and Ag NPs) are commonly used in the fields of electronics, optics, and medicine^8^. Plant extracts are environmentally and economically friendly materials and have been explored in the synthesis of nanoparticles^9^. Turmeric (*Curcuma longa* L.) is widely used as a spice and in cosmetic products. Turmeric is now a popular medicinal plant worldwide and curcumin is the main component of turmeric which functions as a medicine with different properties like anti-oxidant, anti-bacterial, antifungal, anti-parasitic, anti-inflammatory, anti-mutagenic and anti-carcinogenic^10^.

In the ensuing work, aqueous rhizome extracts of the turmeric plant (*Curcuma longa* L.) were used for synthesis of Ag and Au NPs. This plant contains flavonoids, proteins, phenolic compound (such as curcumin) saponins and several mineral elements among others which could reduce the metal ions to their nanoparticles^11,12^.

Plant diversity enables their different applications in material development and altitudinal variation in the synthesis of the aforesaid NPs from plants, which has never been reported yet. In this study rhizome extracts of *C. longa* from different altitudes of Himalayan regions have been investigated for its morphological and phytochemical profiling. The study will equally evaluate the antimicrobial and anticancer properties of the as-developed NPs. It is, however, envisaged that advanced physicochemical properties exhibited by the novel, Ag and Au NPs might influence applications in biomedical, cancer treatment and future medical applications.

## Results

### Qualitative and quantitative screening of phytochemicals

Qualitative phytochemical analysis of *C. longa* rhizome extract collected from different altitudes showed the presence of various active constituents in the aqueous extracts (Table 1). *C. longa* extract contained alkaloids, tannins, flavonoids, carbohydrates, saponins and phenols^13,14^. Quantitative analysis of phytochemicals showed the presence of variable amount of phytoconstituents in the rhizome extracts of three different altitudes (Table 2). The amount of different phytochemicals increased with elevation in altitude^15,16^.

**Table 1:** Screening of phytochemical constituents in *C. longa* rhizomes collected from different altitudes.

S. no.	Compounds	Test performed	Shimla	Mandi	Bilaspur
1	Carbohydrates	Benedict test	+	+	+
2		Molisch test	+	+	+
3	Proteins	Biuret test	+	+	+
4	Flavonoids	Lead acetate	+	+	+
5		Ferric chloride	+	+	+
6	Alkaloids	Wagner’s test	+	+	+
7		Dragendroff test	+	+	+
8	Tannins	Ferric chloride test	+	+	+
9	Saponins	Foam test	+	+	+

**Table 2:** Quantitative estimation of phytochemical content in rhizome extracts of *C. longa* collected from hills Shimla, Mandi and Bilaspur. Values are of mean ± standard error.

S. no.	Phytochemicals	Shimla	Mandi	Bilaspur
1	Carbohydrates (mg/g GLU)	115 ± 3.10	120.46 ± 0.80	130.64 ± 0.14
2	Proteins (mg/g BSA)	148.41 ± 3.87	126.76 ± 3.86	109.12 ± 4.80
3	Phenols (mg/g GAE)	391.14 ± 3.67	345.00 ± 8.59	207.70 ± 75
4	Flavonoids (mg/g RUT)	400.39 ± 7.70	363.37 ± 3.45	220.63 ± 3.66
5	Terpenoids (mg/g LIN)	212.61 ± 5.08	178.94 ± 2.58	162.90 ± 3.370
6	Alkaloids (%)	4.20 ± 0.40	2.50 ± 0.250	1.70 ± 0.20
7	Tannins (mg/ g GAE)	263 ± 1.90	182.49 ± 2.68	164.92 ± 1.90
8	Saponins (mg/g DIO)	138.22 ± 1.40	113.55 ± 1.52	59.15 ± 1.72

### Synthesis of Ag NPs and Au NPs

**Figure 1:**
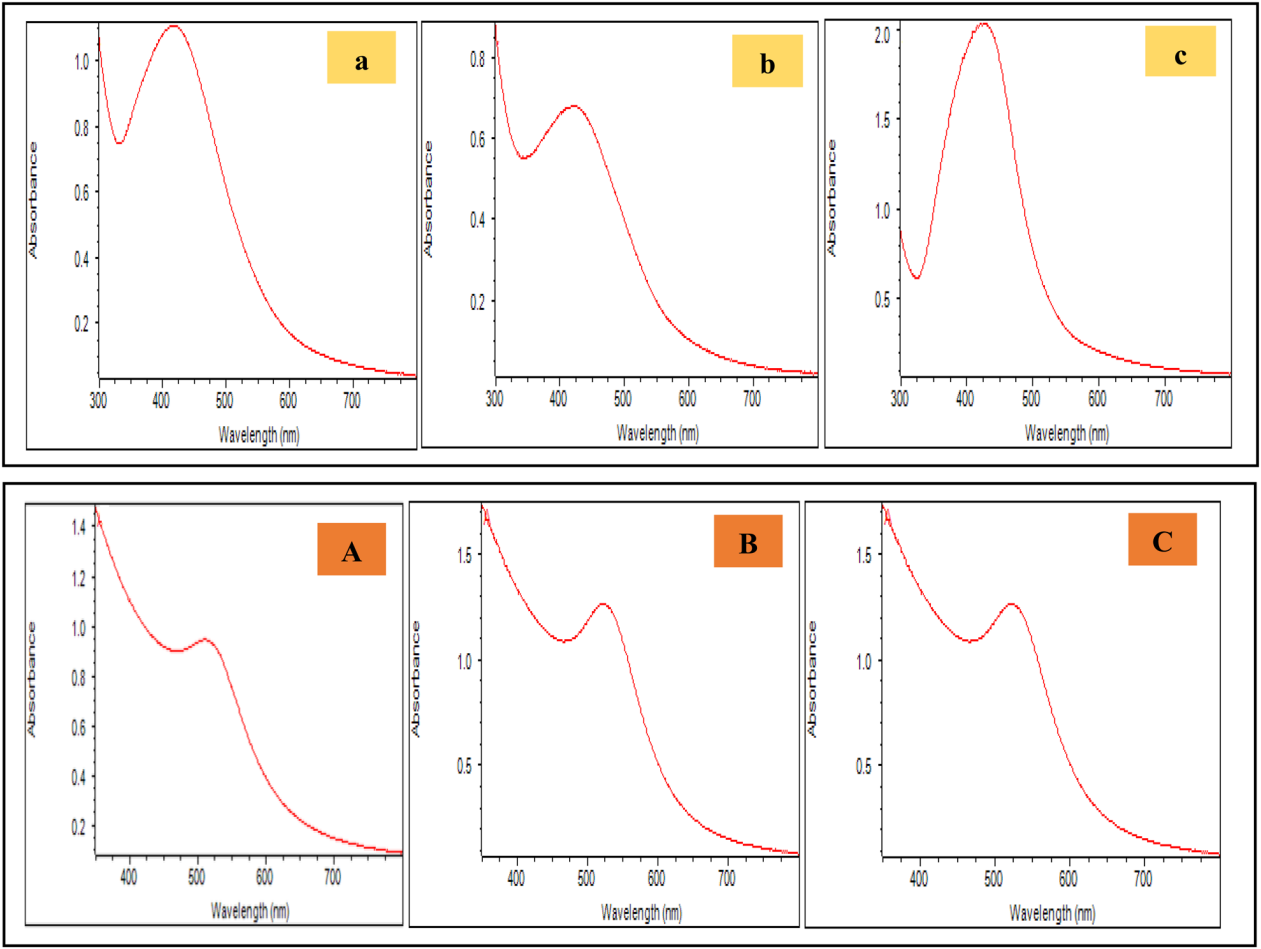
UV–Vis spectra of Ag NPs (upper panel) using rhizome extracts of *C*. *longa* from hills of Shimla (**a**), Mandi (**b**) and Bilaspur (**c**) and Au NPs (lower panel) synthesized using rhizome extracts of *C*. *longa* from hills of Shimla (**A**), Mandi (**B**) and Bilaspur (**C**).

Synthesis of Ag NPs and Au NPs were initially noticed by color changes in the solution (see supplementary Fig. 1 online) and then by the presence of SPR described by UV–Vis spectra in Fig. 1. Various colors were consistent with plasmon bands observed in the figure. As the reaction progressed, colour change in the solution inference the formation of colloidal Ag NPs. Ag NPs synthesized from rhizome extract of hills of Shimla, Mandi and Biaspur divulged absorption peaks at 421 nm, 419 nm and 422 nm, respectively. Due to the nature of the SPR, a wavelength corresponds to that of spherical Ag NPs. Ag NPs prepared from *C. longa* rhizome extract showed UV–Vis spectra at 415 and 420 nm^17,18^.

### Characterization of NPs

In case of Au NPs, bands started appearing at a wavelength of approximately 520 nm (Fig. 1) after 30 min of the reaction. The color change from light yellow to reddish-brown is due to reduction of Au^3+^ to Au^0^, which is characteristic of Au NPs (see supplementary Fig. 1 online). The colour change in the solution indicated the formation of Au NPs (Fig. 1)^19^. Absorption peaks shown by Au NPs synthesized from rhizome extract of Shimla, Mandi and Bilaspur hills was at 520 nm, 519 nm and 518 nm, respectively (Fig. 1)^20,21^.

**Figure 2:**
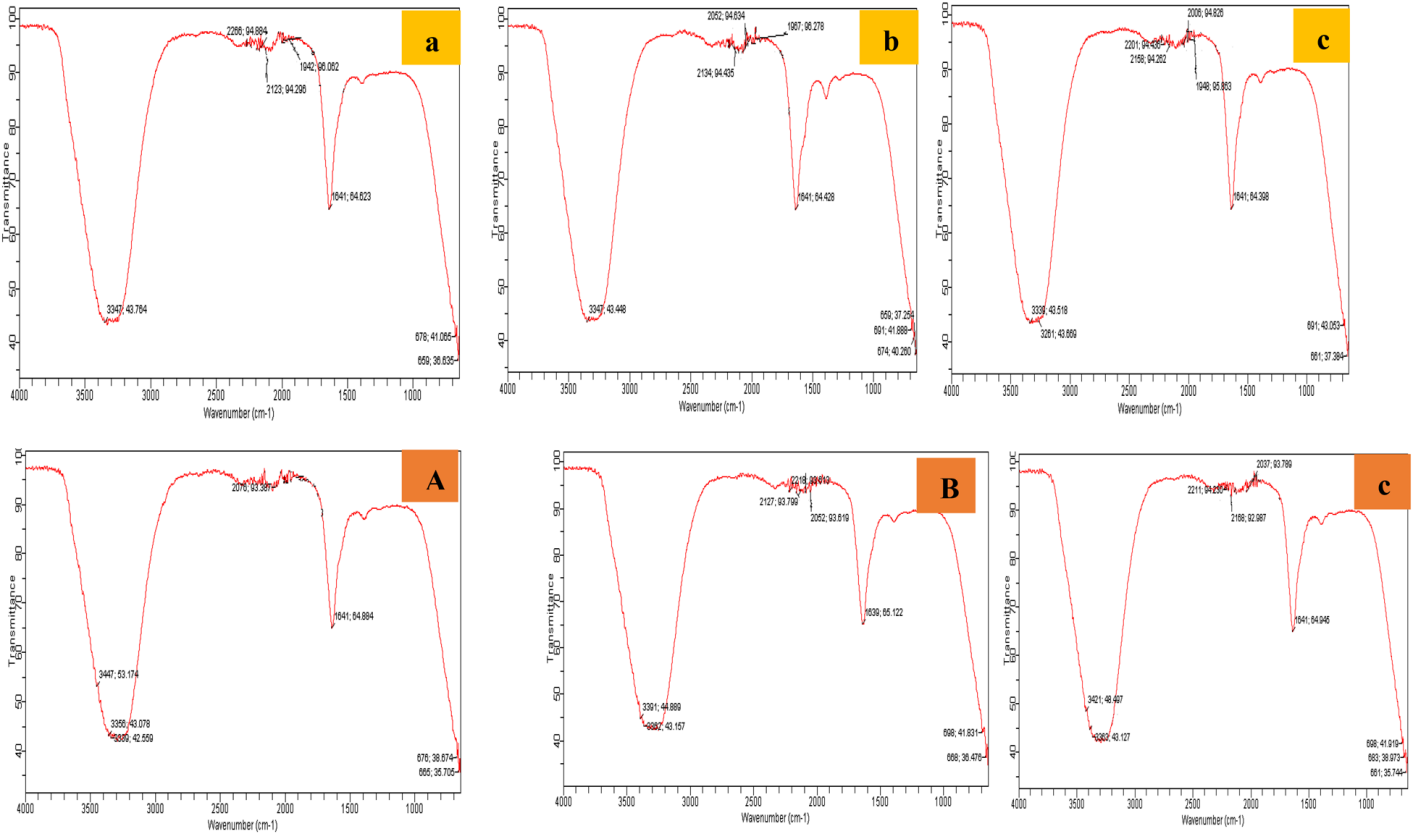
FTIR spectra for Ag NPs (upper panel) using rhizome extracts of *C*. *longa* from hills of Shimla (**a**), Mandi (**b**) and Bilaspur (**c**) and Au NPs (lower panel) synthesized using rhizome extracts of *C*. *longa* from hills of Shimla (**A**), Mandi (**B**) and Bilaspur (**C**).

The plant contains active biomolecules characteristic of functional groups such as amides, hydroxyl, ketones, ethers and alkyls, etc. which have earlier been reported^22^. The three FTIR spectra of Au NPs and Ag NPs synthesized from rhizome extract of Shimla, Mandi and Bilaspur hills have been reported here with possible peaks arising from the biomolecules responsible for reduction, capping and efficient stabilization of the photosynthesized NPs. They all showed similar peak regions (Fig. 2). Various transmission peaks were present at 3347 (O–H group), 1942 (Ag–O), 1641 (C=O group) and 659 showed the presence of primary and secondary amines in the synthesized nanoparticles, 2266 represents C=N stretching. From the assignments, biomolecules like proteins, sugars, esters, carboxylic acids, flavonoids, terpenoids, polyphenolic compounds from the aqueous plant extract may have been responsible for predominantly spherical nanoparticles formation^23^. These compounds could have contributed to the reduction of the metal ions and stability of nanoparticles by donation of electrons. These phytomolecules play a role in Ag and Au NPs synthesis showing greater capping effect by the plant extracts.

**Figure 3:**
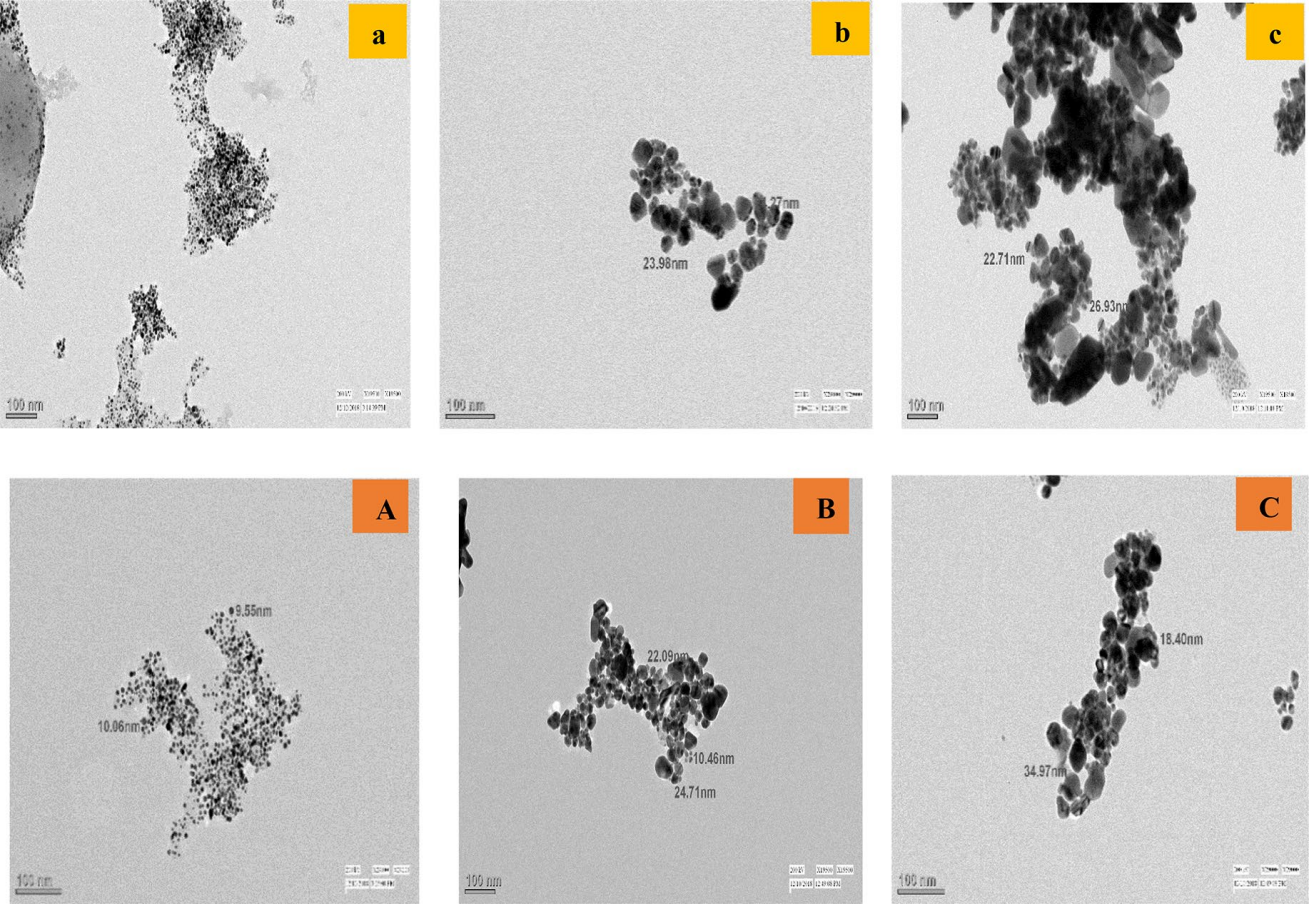
TEM micrographs of Ag NPs (upper panel) synthesized from the rhizome extracts of *C. longa* in hills of Shimla (**a**), Mandi (**b**) and Bilaspur (**c**) and Au NPs (lower panel) synthesized from the rhizome extracts of *C. longa* in hills of Shimla (**A**), Mandi (**B**) and Bilaspur (**C**).

TEM micrographs of the Ag NPs and Au NPs from rhizome extracts of hills Shimla, Mandi and Bilaspur revealed the formation of different sized NPs with spherical morphology. The biochemicals in the aqueous leaf extract were active at capping and stabilizing the nanoparticles. As divulged in Fig. 3 the size of the Ag NPs prepared from rhizome extract of *C. longa* collected from hills Shimla, Mandi and Bilaspur have been found in range of 2–10 nm, 15–35 nm and 25–60 nm, respectively^18,19^. The size range of Au NPs synthesized from *C. longa* rhizome extract collected from Shimla, Mandi and Bilaspur were 2–10, 10–35 and 15–40 nm, respectively (Fig. 3)^21^.

### Antibacterial activity of Ag and Au NPs against pathogenic bacteria

**Figure 4:**
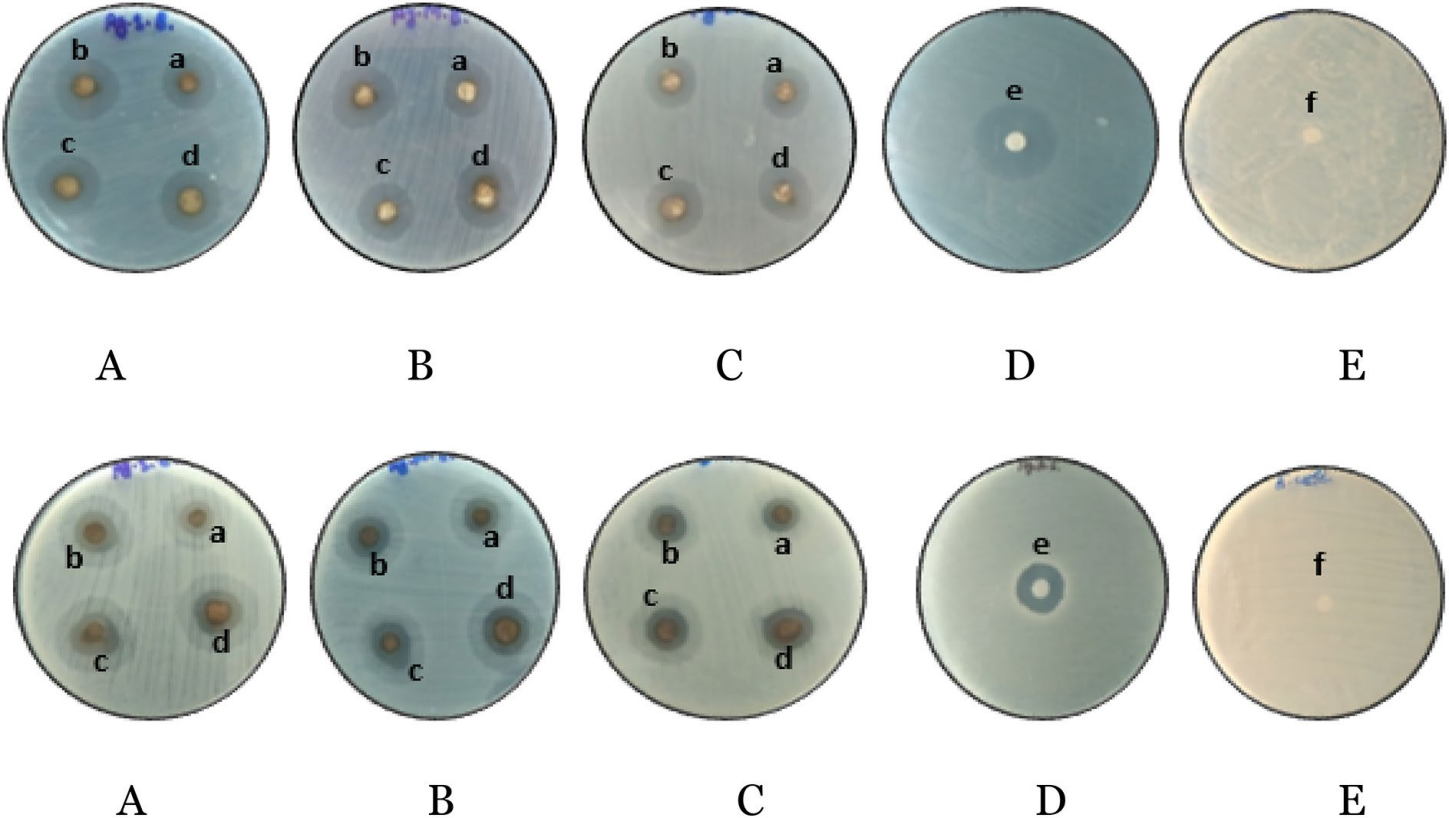
Antimicrobial activity of Ag NPs against: *B. subtilis* (upper panel) and *E. coli* (lower panel) in the hills of Shimla (**A**), Mandi (**B**) and Bilaspur (**C**). Positive control (**D**) (ampicillin), negative control (**E**) (DMSO), where a—150 µg/ml, b—200 µg/ml, c—250 µg/ml, d—300 µg/ml, e—50 µg/ml, f—50 µg/ml.

**Figure 5:**
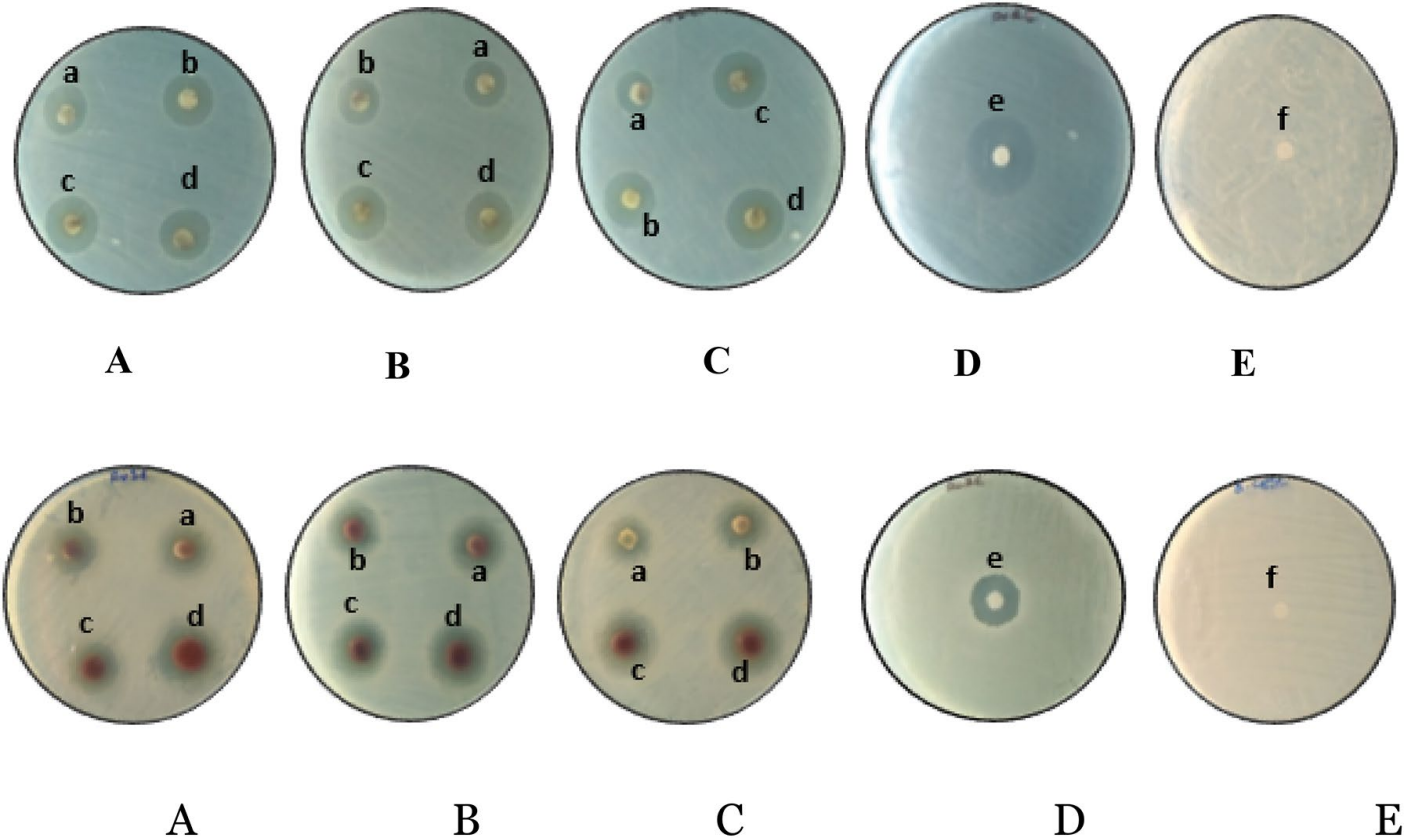
Antimicrobial activity of Au NPs against: *B. subtilis* (upper panel) and *E. coli* (lower panel) in the hills of Shimla (**A**), Mandi (**B**) and Bilaspur (**C**). Positive control (**D**) (ampicillin), negative control (**E**) (DMSO), where a—150 µg/ml, b—200 µg/ml, c—250 µg/ml, d—300 µg/ml, e—50 µg/ml, f—50 µg/ml.

Antibacterial activity of biosynthesized Ag and Au NPs from rhizome extracts of different altitudes have been studied against two pathogenic bacteria using disc diffusion method and zone of inhibition as depicted in (Figs. 4, 5). Discs were loaded with different concentrations—150 µg/ml, 200 µg/ml and 250 µg/ml, 300 µg/ml of Ag and Au NPs, respectively. Maximum zone of inhibition (18.66 ± 1.54) have been observed with *B. subtilis* at 300 µg/ml of Ag NPs synthesized from rhizome extract of Shimla hills. Against *E. coli* maximum zone of inhibition was observed from Ag NPs prepared from rhizome extract of Shimla hills at concentration of 300 µg/ml (Tables 3, 4). The Au NPs via *C. longa* from Shimla hills showed maximum antimicrobial activity at concentration 300 µg/ml against *B. subtilis* and *E. coli* minimum from hills Bilaspur at concentration 150 µg/ml (Table 5). Maximum antibacterial activity has been shown against *P. aeruginosa* (21 mm) and *K. pneumonia*^23^. Antimicrobial activity of the Ag NPs has been found more as compared to the methanolic extract of turmeric and antibiotic^18,24^. Antibacterial activity of *C. longa* rhizome extract and synthesized Au NPs have been studied against two Gram positive bacteria (*B. subtilis, P. aeruginosa*) and two Gram negative bacteria (*S. aureus, E. coli*)^25–27^.

**Table 3:** Antimicrobial activity of Ag NPs of *C. longa* against *E. coli* and *B. subtilis* where (AgS—Ag NPs of sample collected from Shimla, AgM—Ag NPs of sample collected from Mandi, AgB—Ag NPs of sample collected from Bilaspur). The inhibitory zones (in mm) of Ag NPs was measured by Hi media scale at different concentrations.

S. no.	NPs	Bacterial strains	Inhibition zones (in mm)				
			Antibiotic 50 µg/ml	150 µg/ml	200 µg/ml	250 µg/ml	300 µg/ml
1	AgS	*B. subtilis*	20.01 ± 0.57	14.33 ± 0.57	16.01 ± 0.01	17.33 ± 0.57	18.66 ± 1.54
2	AgS	*E. coli*	16.66 ± 0.57	10.66 ± 0.57	12.33 ± 0.57	13.33 ± 0.57	14.33 ± 0.57
3	AgM	*B. subtilis*	19.66 ± 0.57	13.66 ± 1.15	15.66 ± 1.15	17.00 ± 1.00	18.33 ± 0.57
4	AgM	*E. coli*	16.33 ± 0.57	10.33 ± 0.57	11.33 ± 0.57	12.66 ± 0.57	14.00 ± 0.57
5	AgB	*B. subtilis*	16.64 ± 0.01	13.64 ± 0.57	14.66 ± 0.57	16.66 ± 1.15	18.00 ± 1.00
6	AgB	*E. coli*	16.01 ± 0.01	10.00 ± 0.57	10.66 ± 0.57	11.66 ± 0.57	13.33 ± 0.57

**Table 4:** Antimicrobial activity of gold nanoparticles of *C. longa* against *E. coli* and *B. subtilis* where (AuS—Au NPs of sample collected from Shimla, AuM—Au NPs of sample collected from Mandi, AuB—Au NPs of sample collected from Bilaspur). The inhibitory zones (in mm) of Au NPs was measured by Hi media scale at different concentrations.

S. no.	NPs	Bacterial strains	Inhibition zones (in mm)				
			50 µg/ml	150 µg/ml	200 µg/ml	250 µg/ml	300 µg/ml
1	AuS	*B. subtilis*	19.66 ± 0.57	13.00 ± 1.00	14.00 ± 1.00	15.66 ± 0.57	16.68 ± 0.57
2	AuS	*E. coli*	16.01 ± 0.01	10.66 ± 0.57	11.00 ± 0.57	11.66 ± 0.57	13.33 ± 0.57
3	AuM	*B. subtilis*	20.01 ± 0.01	11.66 ± 0.57	13.66 ± 0.57	14.66 ± 0.57	16.00 ± 0.57
4	AuM	*E. coli*	16.66 ± 0.57	10.33 ± 0.57	10.66 ± 0.57	11.33 ± 0.57	12.33 ± 0.57
5	AuB	*B. subtilis*	19.66 ± 0.57	11.44 ± 0.57	13.33 ± 0.57	14.33 ± 0.57	15.33 ± 0.57
6	AuB	*E. coli*	16.01 ± 0.01	10.00 ± 0.57	10.33 ± 0.57	11.00 ± 0.57	12.00 ± 0.57

**Table 5:** Comparison of antimicrobial activity of extract and silver and gold nanoparticles and plant extract from Shimla [inhibition in mm (300 µg/ml)]. ES—rhizome extract of C. longa collected from Shimla, AgS—silver nanoparticles of sample collected from Shimla, AuS—gold nanoparticles of sample collected from Shimla.

S. no.	NPs	Bacterial strains	Inhibition zones
1	ES	*B. subtilis*	10.66 ± 0.57
2	ES	*E. coli*	9.66 ± 0.57
3	AgS	*B. subtilis*	18.66 ± 1.54
4	AgS	*E. coli*	14.33 ± 0.57
5	AuS	*B. subtilis*	16.68 ± 0.57
6	AuS	*E. coli*	13.33 ± 0.57

### Anticancer activity of Ag and Au NPs

**Figure 6:**
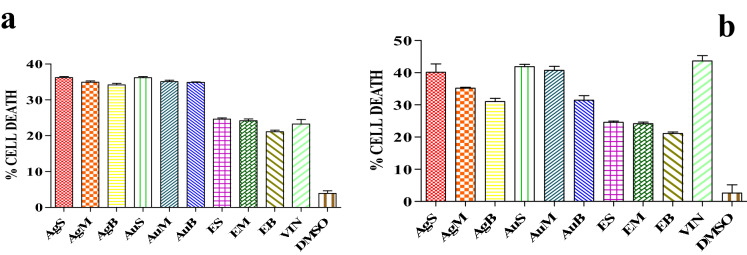
Percentage cell death of (**a**) A549 cells and (**b**) PC3 cells after treatment with Ag and Au NPs for 24 h.

The Ag and Au NPs prepared from rhizome extract of *C. longa* from different altitudes revealed anticancer activity against A549 (lung adenocarcinoma) (see supplementary Fig. 2 online) and PC3 (prostate cancer) cell lines (see supplementary Fig. 3 online). A comparison among results of percentage cell death of silver and Au NPs against A549 and PC3 cell line (VIN—vincristine sulphate (+ ve control), DMSO (− ve control) has been shown in Table 6. The Ag NPs synthesized from rhizome extract of *C. longa* from Shimla vicinity demonstrated highest cytotoxic activity (40%) against A549 cells at concentration of 100 µg/ml. Maximum cytotoxic activity of Au NPs against PC3 cells was observed in the sample prepared from Shimla hills (36%) followed by the sample prepared from Mandi hills (35%) and 34% for hills Bilaspur (Fig. 6). Similar results has been reported whereby anti-cancer efficacy of bio functionalized Au and Ag using different plant extracts of guava and clove checked against four different cell lines^28–31^.

**Table 6:** Results of percentage cell death of silver and Au NPs against A549 and PC3 cell line (VIN—vincristine sulphate (+ ve control), DMSO (− ve control), AgS—Ag NPs of sample collected from Shimla hills, AgM—Ag NPs of sample collected from Mandi hills, AgB—Ag NPs of sample collected from Bilaspur hills. silver AuS—Au NPs of sample collected from Shimla hills, AuM—Au NPs of sample collected from Mandi hills, AuB—Au NPs of sample collected from Bilaspur hills, ES—*C. longa* extract of Shimla hills, EM—*C. longa* extract of Mandi hills, EB—*C. longa* extract of Bilaspur).

Sample	Percentage cell death	
	A549	PC3
VIN	43.66 ± 4.67	23.34 ± 2.26
DMSO	2.60 ± 2.56	3.90 ± 0.76
AgS	40.16 ± 2.58	36.21 ± 0.32
AgM	35.17 ± 0.30	34.92 ± 0.16
AgB	31.04 ± 1.00	34.18 ± 0.43
AuS	41.89 ± 0.70	36.19 ± 0.34
AuM	40.72 ± 0.25	35.09 ± 0.39
AuB	31.49 ± 1.36	34.89 ± 0.11
ES	24.60 ± 0.36	24.60 ± 0.36
EM	24.16 ± 0.50	24.16 ± 0.50
EB	21.12 ± 0.43	21.12 ± 0.43

## Materials and methods

### Materials

Silver nitrate (AgNO_3_) and gold (III) chloride hydrate (HAuCl4·xH_2_O) were obtained from Loba Chemie. Rhizomes of turmeric were collected from three hills of Himachal Pradesh. Identification was confirmed by a plant taxonomist at Botanical Survey of India, Dehradun. Rhizomes were washed to remove sand and debris and dried at room temperature under air for 1 week and powdered. An aqueous extract was prepared by adding 2.5 g of rhizome powder in 100 ml of distilled water and then kept inside orbital shaker for 48 h at room temperature. The filtered solution was cooled, dried and used for synthesis of NPs. The extract solution used for all the syntheses was stable but when in combination with the metal salts, changes in color were noticed^9^.

### Qualitative and quantitative screening of phytochemicals

Aqueous extracts of rhizomes of *C. longa* from different altitude were used for screening of phytochemicals. The presence of different phytochemicals such as carbohydrates, proteins, flavonoids, alkaloids, tannins, saponins in the rhizome extracts of *C. longa* from different sites were studied. Quantitative analysis was done to analyze the presence of variable amount of phytochemicals in the rhizome extract of *C. longa* collected from different altitudes.

### Synthesis of Ag NPs and Au NPs

For biosynthesis of Au NPs, about 5 mg of rhizome extract of *C. longa* was mixed with 30 ml, 0.06 M Na_3_ Cit solution^19^. Afterward, freshly prepared 5 ml, 0.005 M HAuCl_4_ solution was added drop wise at 30 °C for 30–40 min on a magnetic stirrer. The solution was sampled at different intervals and appearance of surface plasmon resonance has been monitored by use of an UV–Vis spectroscopy (Thermo Fisher Scientific Evolution 160 UV–Vis spectrometer). There was a gradual color change during the course of the reaction. A similar procedure was applied for synthesis of Ag NPs by use of AgNO_3_ salt. In the case, about 5 mg of rhizome extract of *C. longa* was mixed with 30 ml, 0.06 M Na_3_ Cit solution^16^. After that freshly prepared 5 ml, 0.02 M AgNO_3_ solution was added drop wise at temperature 40–60 °C for 30–40 min on magnetic stirrer. There were periodic changes in color due to formation of nanoparticles as shown in Fig. 1. The color changes were confirmed by the presence of surface plasmon resonance (SPR) band obtained in UV–Vis spectra.

### Characterization of NPs

The appearance and stability of Ag and Au NPs were monitored and characterized by use of absorbance peaks (UV–Vis spectrophotometer)^16^.

The particle size and morphological aspects of synthesized NPs were illustrated via transmission electron microscopy (TEM)^8^. TEM was performed by use of Philips CM10 transmission electron microscope operating at 120 kV. The system was fitted an intensified video camera to assist the alignment and a slow scan CCD (charge-coupled device) camera. Final images were recorded on CCD. Fourier transform infrared (FTIR) spectra of NPs were determined by use of an ATR Cary 630 infrared spectrometer operated at a frequency range of 4,000–650 cm^−1^ at a resolution of 16 cm^−1^. Prior to the Fourier transform infrared spectroscopy (FTIR) analysis, the prepared NPs solution was centrifuged and dried.

### Antibacterial activity of Ag and Au NPs against pathogenic bacteria

Biosynthesized Ag and Au nanoparticles were studied for antibacterial activity against pathogenic bacteria using disc diffusion method^13^. The test organisms used were *Bacillus subtilis* (Gram positive) and *Escherichia coli* (Gram negative). Nutrient Agar was used as the media for the culturing of bacterial strains. Various concentrations of nanoparticles synthesized from rhizomes of *C. longa* from different altitudes have been tested against bacterial culture. Strains were swabbed on the surface of the Sabouraud agar plates and discs were prepared from Whatman No. 1 filter paper. For the sake of comparison, the anti-bacterial activities, blank disc impregnated with DMSO was used. Diverse concentrations Ag and Au nanoparticles (150, 200, 250 and 300 µg/ml) were added on the disc and the plates were incubated at 37 °C for 24 h. The antimicrobial potency of the test samples were measured by determining the diameter of the zones of inhibition in millimeter.

### Anticancer activity of Ag and Au NPs

Anticancer activity of Ag and Au NPs was tested against two cancer cell lines i.e. A549 (lung adenocarcinoma) and PC3 (prostate cancer). Cell lines were treated with different Ag and Au NPs synthesized from *C. longa* rhizomes from different altitudes at 100 µg/ml concentration^14^.

## Discussion

In this study qualitative analysis of phytochemicals revealed the presence of different phytochemicals including carbohydrates, proteins, flavonoids, alkaloids, tannins and saponins. Our results are in agreement with the previous work done^17,29,30^ whereby they studied phytochemicals in the rhizomes of *C. longa* and reported the presence of saponins, steroid, tannin, anthocyanin, coumarin, emodins, protein, amino acid, flavonoids, diterpenes, phytosterol, phenol, phlobatannin, leucoanthocyanin, anthroquinone, chalcones, glycosides and carbohydrates with different solvents (acetone, methanol, ethanol and chloroform). The presence of various phytochemicals (carbohydrates, proteins, flavonoids, tannins, alkaloids, saponins and glycosides) in different solvents (petroleum ether, benzene, chloroform, methanol and water) from *C. longa* rhizome extract^32,37^. Results from the quantitative analysis of aqueous extract of *C. longa* rhizomes reported maximum amount of carbohydrates, proteins, in the rhizomes collected from district Bilaspur and tannins, phenols and saponins flavonoids, terpenoids and alkaloids were found highest in the sample collected from district Shimla (highest altitude).

UV–Vis spectrophotometric analysis of silver nanoparticles synthesized from rhizome extract of *C. longa* from hills of Shimla, Mandi and Bilaspur showed maximum peak of absorption at 421 nm, 419 nm and 422 nm, respectively whereby for gold nanoparticles maximum peak of absorption were observed at 520 nm for Shimla, 519 nm for Mandi and for Bilaspur at 518 nm. Transmission electron microscopy (TEM) showed the formation spherical silver and gold nanoparticles in the size range of 2–60 nm. Smallest size nanoparticles were formed from the rhizome extract of *C. longa* collected from higher altitudes, where maximum amount of various phytochemicals (proteins, phenols and alkaloids etc.) were observed, which showed important role in controlling the shape and size of the nanoparticles. It was studied that functional groups such as phenolic and alkaloids are responsible for capping, stabilizing and reduction of nanoparticles^20,33,35^. The reduction mechanism also capable to control the size and stability of the nanoparticles produced from plant extracts^34^. Polysaccharides have many functionalities including hydroxyl groups and a hemiacetal reducing end that are capable of reducing precursor salt. The oxidation of polysaccharides hydroxyl groups to carbonyl groups plays important role in the reduction of gold salts^35,38^.

The silver and gold nanoparticles synthesized from rhizome extract of *C. longa* from different altitudes showed maximum antimicrobial activity against *B. subtilis* and *E. coli* from district Shimla at concentration 300 μg/ml and minimum activity was observed at concentration 150 μg/ml from district Bilaspur. The gold nanoparticles of *C. longa* from district Shimla showed maximum antimicrobial activity at concentration 300 μg/ml against *B. subtilis* and *E. coli* minimum from district Bilaspur at concentration 150 μg/ml. This could be due to smaller size of nanoparticles and presence of variable amount of phytochemicals in the rhizome extract of *C. longa* collected from Shimla hills. Size and shape dependent antibacterial activity of silver nanoparticles against two Gram-negative bacteria *Pseudomonas aeruginosa* and *Escherichia coli* was checked by Amin et al.^20^. Antibacterial activity of the smallest-sized spherical silver nanoparticles was observed maximum against both bacterial strains as compared to the triangular and larger spherical shaped silver nanoparticles.

The maximum anticancer activity of silver and gold nanoparticles against A549 and PC3 cell lines was observed in the nanoparticles synthesized from rhizome extract of *C. longa* collected from district Shimla. This can be due the presence of more amount of phenols, flavonoids and tannins etc. in the rhizome extract and smaller size of the nanoparticles. Similarly synthesized bio functionalized gold and silver nanoparticles using different plant extracts of guava and clove and checked anti-cancer efficacy against four different cancer cell lines human colorectal adenocarcinoma, human kidney, human chronic myelogenous, leukemia, bone marrow, and human cervix^35,36,38^. They concluded that flavonoids functionalized gold nanoparticles synthesized using aqueous clove buds extract are more effective than guava leaf extract towards anti-cancer activities.

## Conclusions

Medicaments play important role in drug development as plants produce a wide range of phytochemicals. Herbal drugs are prescribed widely because of their effectiveness and no side effect in clinical experience^33,37^, although their biologically active compounds are unknown. The ensuing endeavor present an evaluation of *Curcuma longa* and antimicrobial and anticancer activity of Ag and Au NPs synthesized from rhizome extract of plant^20,34^ collected from varied altitudes. The nanoparticles demonstrated an intense antibacterial activity against *E. coli* and *B. subtilis.* Anticancer activity of the aforesaid nanoparticles has been checked against A549 and PC3 cell lines. The synthesized nanoparticles revealed variation in size with elevated altitude. Small size of nanoparticles has been reported at higher altitude (Shimla hills). Various phytochemicals including flavonoids, phenols, tannins, and terpenoids were found in maximum amount in rhizome extracts of higher altitude. The greater surface stabilization effect as a result of maximum phytochemicals presence could be a possible reason for reduction in the size of nanoparticles^30^. Ag and Au nanoparticles both inhibit the bacterial growth. The Ag nanoparticles synthesized from rhizome extract of hills Shimla with higher altitude showed maximum antimicrobial activity. These nanoparticles revealed percentage cell cytotoxicity against A549 and PC3 cell lines. *Curcuma longa* has been used in traditional medicine as a household remedy for different diseases in many parts of the world. Nanoparticles prepared from the rhizome extract of *C. longa* can be used to develop many medicines and may be supportive tool to cure many diseases. The use of such eco-friendly nanoparticles as an anticancer agent in medical applications, render this method potentially exciting for a large-scale synthesis of other inorganic materials (nanomaterials). The effect of anticancer activity of rhizome extract of *Curcuma* *longa *can be enhanced via formation of the aforesaid nanoparticles by the projected method. Results revealed that Ag and Au NPs demonstrated effective anticancer activity en route for cancer cell lines. Size variation of nanoparticles varies with altitude and has publicized immense variation in cancer activity. Therefore, in conclusion we out to inference that altitude has major effect on size of nanoparticle and the same nanoparticles were more effective in cancer inhibition than the large sized NPs.

## Supplementary information

Supplementary file1 (DOCX 3990 kb)

## Data Availability

All dates generated and analyzed during this study are included in this paper.

## Abbreviations

µg: Micro gram
µl: Micro litre
AgB: Silver nanoparticles of extract of Bilaspur hill
AgM: Silver nanoparticles of extract of Mandi hill
AgS: Silver nanoparticles of extract of Shimla hill
Au: Gold
AuB: Gold nanoparticles of extract of Bilaspur hill
AuM: Gold nanoparticles of extract of Mandi hill
AuS: Gold nanoparticles of extract of Shimla hill
DMSO: Dimethyl sulfoxide
EB: Extract of Bilaspur hill
EM: Extract of Mandi hill
ES: Extract of Shimla hill
GAE: Gallic acid equivalents
GLU: Glucose
LIN: Linalool
MIC: Minimum inhibitory concentration
MTT: 3-(4,5-Dimethylthiazol-2-yl)-2,5-diphenyltetrazoliumbromide
RUT: Rutin
VIN: Vincristine sulphate
