# Supplementary material for: Unveiling antimicrobial and anticancerous behavior of AuNPs and AgNPs moderated by rhizome extracts of Curcuma longa from diverse altitudes of Himalaya
Source: Sci Rep. 2020 Jul 2;10:10934. doi: 10.1038/s41598-020-67673-4 (PMC7331668; doi:10.1038/s41598-020-67673-4)
Supplement: Supplementary file 1 — Supplementary file1 (DOCX 3990 kb) [file 41598_2020_67673_MOESM1_ESM.docx]

# **Unveiling antimicrobial & anticancerous behavior of AuNPs & AgNPs moderated by rhizome extracts of *Curcuma longa* from diverse altitudes of Himalaya**

# Mamta Sharma*^1,^, Monika ^1^, Pankaj Thakur*^2,3^ , Reena V. Saini^4^, Rajesh Kumar^5^, Enza Torino^6^

*^1^School of Biological and Environmental Sciences, Shoolini University, Solan, India-173212,* [*mamta131526@gmail.com*](mailto:mamta131526@gmail.com)

*^2^School of Chemistry, Shoolini University, Solan, India-173212.* [*chempank@gmail.com*](mailto:chempank@gmail.com)

*^3^Himalayan Center of Excellence in Nanotechnology, Shoolini University, Solan, India-173212.*

*4 School of Biotechnology and Applied Sciences, Shoolini University.*

*5 School of Physics and Materials Sciences, Shoolini University.* [*rajeshsharma@shooliniuniversity.com*](mailto:rajeshsharma@shooliniuniversity.com)

*^6^ Center for Advanced Biomaterials for Healthcare, Italian Institute of Technology, Naples, Italy-80125.*

**Supplementary Figures:**


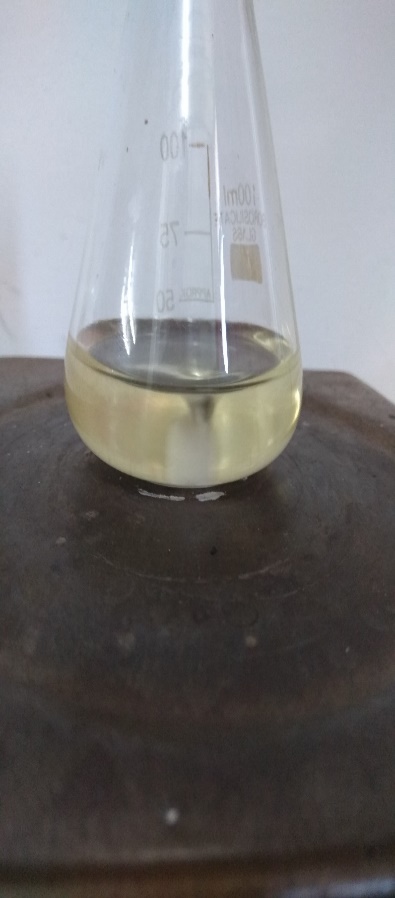

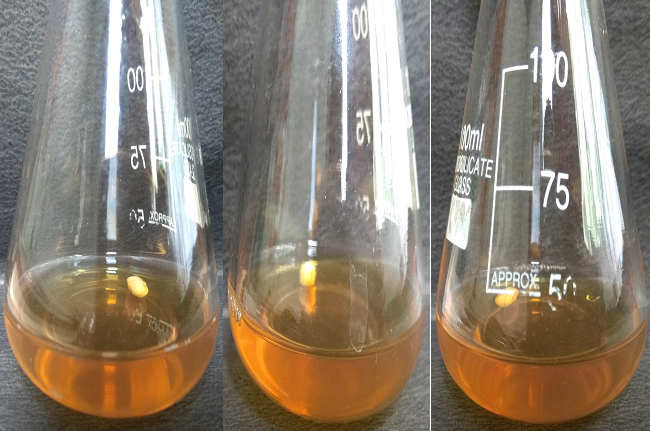

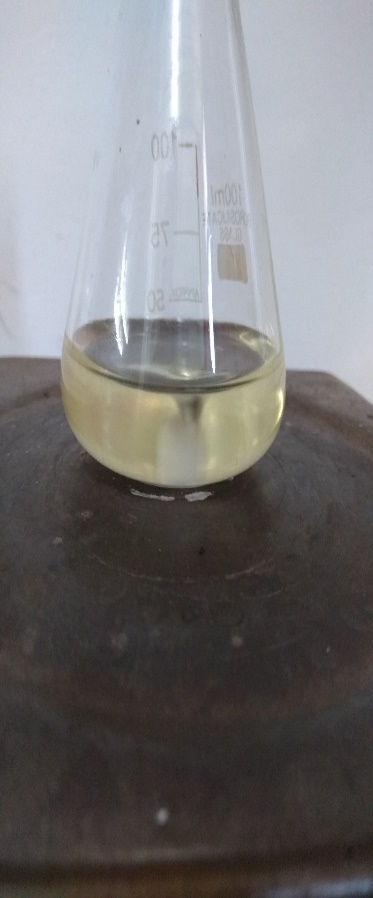

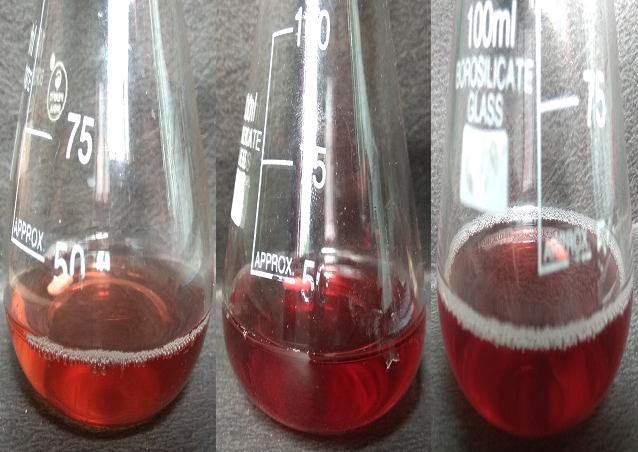


**Ag+Plant extract+Na_3_Cit**

**AgNPs**

**30 min**

**Au+Plant extract+Na_3_Cit**

**AuNPs**

**30 min**

**Fig.1. Illustration of color changes during preparation of NPs**


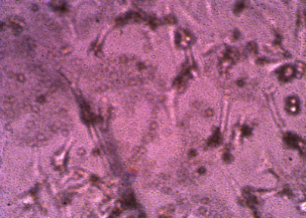

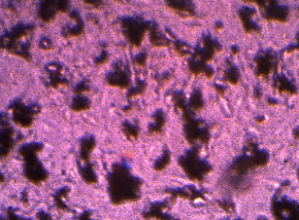

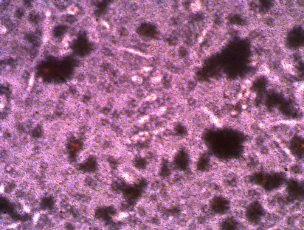


**AuB**

**AgB**

**AgM**

**AgS**


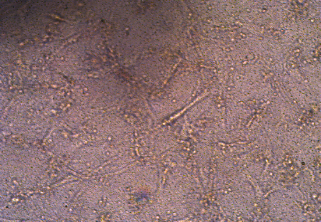

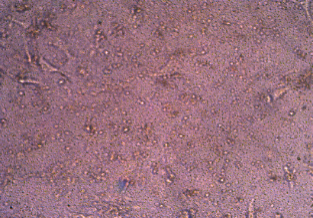

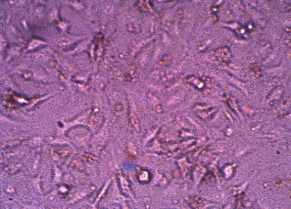


**EB**

**EM**

**AuM**

**AuS**


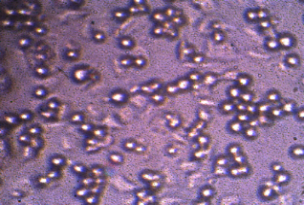

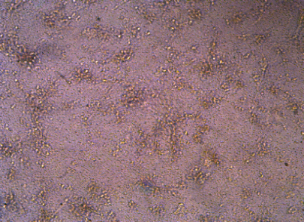

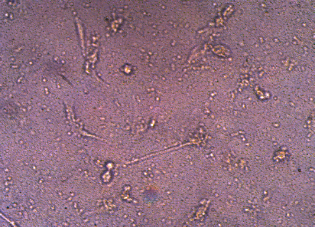

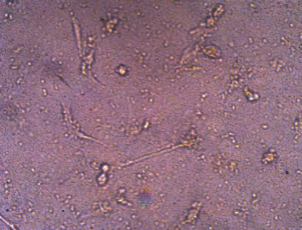


**VIN**

**ES**

**Fig.2. Morphological analysis of anticancer activity of Ag NPs & Au NPs against A549 cell line, (VIN- Vincristine sulphate, AgS- Ag NPs of sample collected from Shimla hills, AgM- Ag NPs of sample collected from Mandi hills, AgB- Ag NPs of sample collected from Bilaspur hills. AuS- AuNPs of sample collected from Shimla, AuM- AuNPs of sample collected from Mandi, AuB- AuNPs of sample collected from Bilaspur, ES- *C. longa* extract of Shimla hills, EM- *C. longa* extract of Mandi hills, EB- *C. longa* extract of Bilaspur hills).**


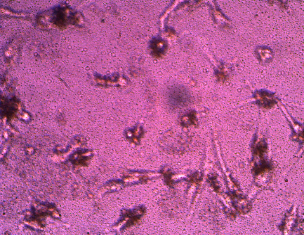

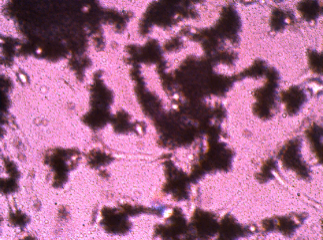

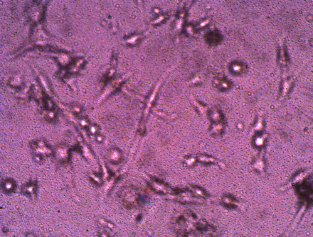


**AgB**

**AgM**

**AgS**


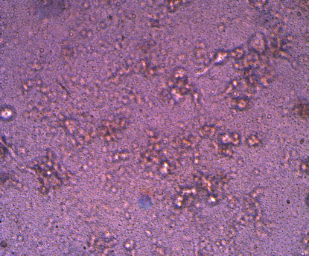

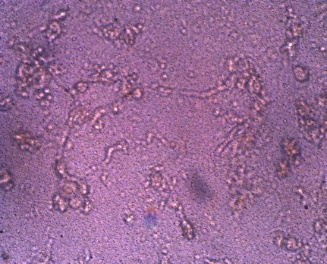

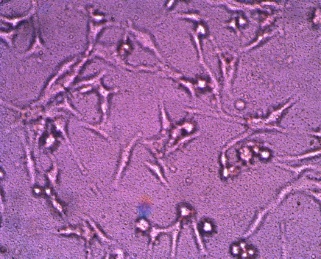


**AuB**

**AuM**

**AuS**


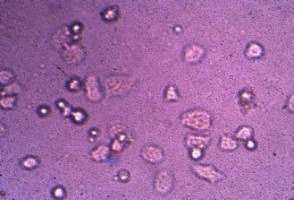

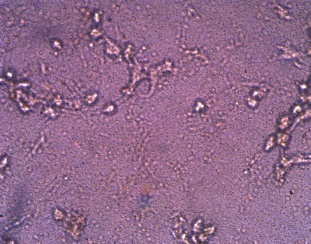

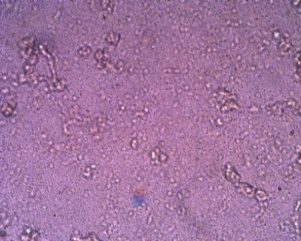

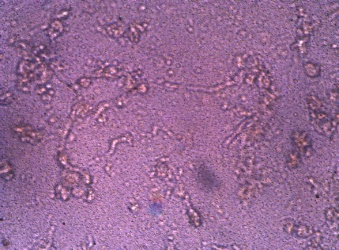


**VIN**

**EB**

**EM**

**ES**

**Fig.3. Morphological analysis of anticancer activity of Ag NPs & Au NPs against PC3 cell line, (VIN- Vincristine sulphate, AgS- Ag NPs of sample collected from Shimla hills, AgM- Ag NPs of sample collected from Mandi hills, AgB- Ag NPs of sample collected from Bilaspur hills. AuS- AuNPs of sample collected from Shimla, AuM- AuNPs of sample collected from Mandi, AuB- AuNPs of sample collected from Bilaspur, ES- *C. longa* extract of Shimla hills, EM- *C. longa* extract of Mandi hills, EB- *C. longa* extract of Bilaspur hills).**
